# Alpha Particle Detection Using Alpha-Induced Air Radioluminescence: A Review and Future Prospects for Preliminary Radiological Characterisation for Nuclear Facilities Decommissioning

**DOI:** 10.3390/s18041015

**Published:** 2018-03-28

**Authors:** Anita J. Crompton, Kelum A. A. Gamage, Alex Jenkins, Charles James Taylor

**Affiliations:** 1 Engineering Department, Lancaster University, LA1 4YW Lancaster, UK; c.taylor@lancaster.ac.uk; 2 School of Engineering, University of Glasgow, G12 8QQ Glasgow, UK; kelum.gamage@glasgow.ac.uk; 3 Characterisation, Inspection & Decontamination Group, Sellafield Ltd., CA20 1PG Cumbria, UK; alex.jenkins@sellafieldsites.com

**Keywords:** alpha detection, alpha-induced air radioluminescence, alpha imaging, nuclear decontamination and decommissioning

## Abstract

The United Kingdom (UK) has a significant legacy of nuclear installations to be decommissioned over the next 100 years and a thorough characterisation is required prior to the development of a detailed decommissioning plan. Alpha radiation detection is notoriously time consuming and difficult to carry out due to the short range of alpha particles in air. Long-range detection of alpha particles is therefore highly desirable and this has been attempted through the detection of secondary effects from alpha radiation, most notably the air-radioluminescence caused by ionisation. This paper evaluates alpha induced air radioluminescence detectors developed to date and looks at their potential to develop a stand-off, alpha radiation detector which can be used in the nuclear decommissioning field in daylight conditions to detect alpha contaminated materials.

## 1. Introduction

**Figure 1:**
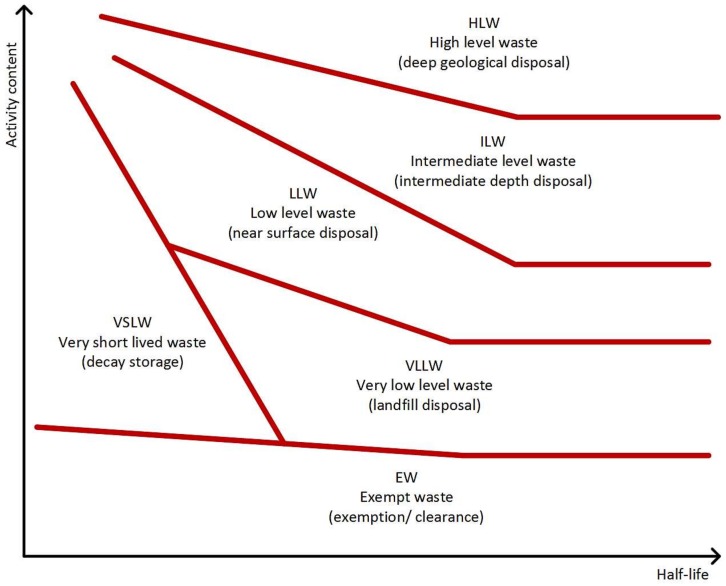
The different delineated areas show nominal waste classification according to activity level and half-life. Half-lives range from seconds to millions of years, with ‘short lived’ considered to be less than approximately 30 years. Reproduction of the conceptual illustration of the waste classification scheme diagram, from: International Atomic Energy Agency, Classification of Radioactive Waste, IAEA Safety Standards Series, No. GSG-1, IAEA, Vienna, 2009 [2]. Reproduced with permission from IAEA.

Since its inception in the 1940s firstly as a means to produce plutonium for weapons and later for energy generation, the UK nuclear industry has as a consequence of operations seen radioactive contamination of its facilities across the UK. This is an unavoidable consequence of nuclear processes and an anticipated phenomenon. At the end of their useful life, these facilities require decommissioning and clean up to remove hazardous substances in order that the site can be repurposed or reused. This produces significant quantities of waste, which is forecast to reach a total of 4.7 million tonnes over the next 100 years [1]. This waste falls into several categories depending on the type, levels, activity, half-life, etc. of radioactivity of the waste including: very short lived waste (VSLW); very low level waste (VLLW), low level waste (LLW), intermediate level waste (ILW) or high level waste (HLW); and waste which does not exhibit any radioactive contamination (EW—Exempt Waste) (see Figure 1).

How the different types of waste are collected and treated differs, from the personal protective equipment (PPE) required by personnel, to the process of collection and processing, and the storage of the waste, all of which have associated cost implications. In general HLW is much more costly to deal with than LLW, which is in turn more costly than uncontaminated waste. This is due to the increasing safety precautions required with increased activity: for example lower safe exposure times for staff meaning shorter working times, increased amounts of PPE required, pre storage decontamination and other treatment, greater shielding required of storage receptacles and facilities, specialist equipment e.g., robots in areas too active for human intervention.

It is therefore important for financial and safety reasons that plant and equipment is correctly characterised prior to decommissioning taking place in order that a suitable, efficient and safe plan for the removal and storage of waste can be drawn up and implemented. As part of this characterisation process, the identification and location of alpha radiation emitting sources is an important element. Plutonium contaminated materials which are almost exclusively alpha emitters are widespread in nuclear reprocessing facilities, yet these are difficult to detect by non-destructive or passive detection methods posing a problem for characterisation efforts.

This paper looks at existing alpha particle detection methods, particularly the detection of alpha particles through alpha-induced air-radioluminescence. It attempts to draw together the existing research on this subject and to lay out a path to progress the understanding and capability in this area based on the foundation of work carried out to date. The work is primarily focused on the research into and application of alpha detection technology for nuclear decommissioning, although it is possible that there could be applications for other areas such as nuclear safeguards and security.

## 2. Alpha Radiation

Alpha particles are comprised of two protons and two neutrons. They have a relatively strong positive charge and therefore interact strongly with molecules in the air as they are emitted from a radioactive source, transferring their energy within a range of a few centimeters depending on their initial energy. Their atomically large mass and charge also means that they are easily stopped in solid matter, for example by a sheet of paper or skin. Although the least penetrating form of radiation, if ingested alpha particles cause the most internal damage relative to absorbed dose due to their high linear energy transfer, making them hazardous to humans. Despite the biological hazard increasing from gamma to beta to alpha radiation, there are correspondingly less detectors available, and as some contamination isotopes may only be alpha emitters, this makes a new way to detect alpha more important [3].

**Figure 2:**
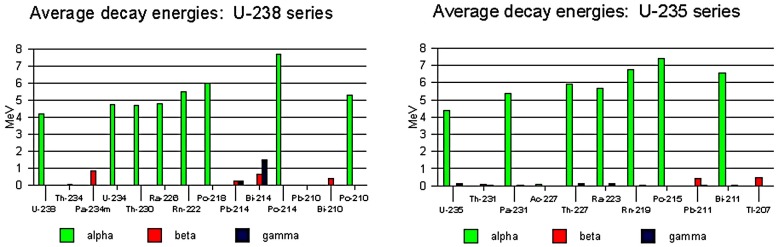
Average decay energies of U-238 and U-235 series. Source: WISE Uranium Project [7].

Actinides, to which group the main isotopes found in nuclear applications belong, are primarily alpha emitters, giving off relatively weak beta and gamma radiation, which is also of low energy. Figure 2 shows a comparison of alpha, beta and gamma emissions from two uranium isotopes widely found in the nuclear industry [4]. The main isotopes found in nuclear applications, which are predominantly alpha emitters, are uranium-235, uranium-238, plutonium-238, plutonium-239 and americium-241 [5]. Alpha emissions are more likely from trans-uranic elements, those with a greater atomic number than uranium, for example Pu and Am, where the high atomic mass makes the isotopes unstable. Technology available at present is less effective for characterising actinides [6], which as the primary isotopes in nuclear applications, has implications for the nuclear industry, making advances in alpha detection highly desirable.

Due to the short range of alpha particles, traditional detectors which require direct interaction with the alpha particles must be used in very close proximity to a contaminated surface, around 1 cm [4]. This makes detecting alpha radiation time consuming, taking in the order of hours for one room [8]. It also requires the use of PPE to prevent ingestion by personnel in close proximity to alpha sources, including the danger of inhalation if disturbed, contaminated material becomes airborne. It may also be necessary to protect against exposure to other types of radiation which may also be present. Samples are taken from suspected areas and analyzed in a vacuum for complete characterisation [4] which can take significant time and cost [9].

Due to these difficulties, and those in the development of direct alpha particle detectors, a new way to detect alpha radiation is being sought which can be accomplished at a distance using secondary effects, for example alpha-induced air-radioluminescence. In this paper the authors review such alpha detection techniques and discuss further improvements and prospects for nuclear decommissioning applications.

## 3. Alpha-Induced Air-Radioluminescence

The most prevalent method of detecting alpha radiation at a distance is through the detection of the UV photons emitted by nitrogen after receiving energy from alpha particle emissions. After emission from a source, an alpha particle’s energy is transferred directly and via secondary electrons, to molecules with which they interact. When these molecules relax they may emit an Ultraviolet (UV) photon. Although the alpha particle and the secondary electrons they generate through ionisation have a range of only a few centimeters (depending on their energy), UV photons have a much longer mean free path (MFP) in air than alpha particles and therefore can be detected at a much greater distance from the source than a traditional detector would allow.

**Figure 3:**
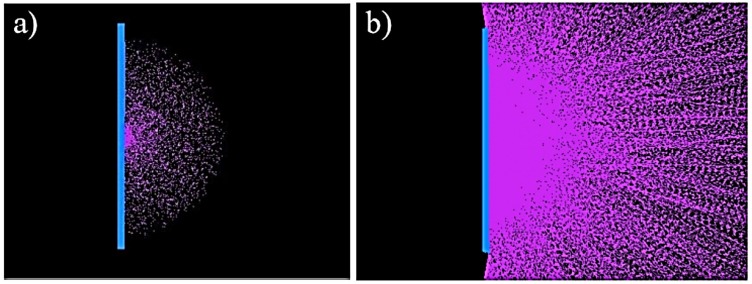
Model of: (**a**) Radioluminescence photons induced by alpha particles showing the hemisphere in which they are initially created by the alpha particles; (**b**) Showing the random directions in which the photons are emitted from the hemisphere in (**a**) and their longer path length—Using FRED Optical Engineering Software (Photon Engineering LLC) [11]. Reprinted with permission from the author.

Researchers found the range of alpha particles with energy of 5.1 MeV to be 38 mm in air, with the area of highest intensity of radioluminescence scintillation within a radius of 10 mm from the source [10]. Others found by simulation that the range within which the energy of the alpha particle was transferred was approximately 5 cm for a 6.1 MeV source [8]. For a point source in space the zone of alpha particles would be a sphere with a radius of the range of the alpha particle emissions. For a point source on a surface this would be a hemisphere with the same radius, see Figure 3, [11,12].

The photons generated, similarly to the alpha particles themselves, form a hemispherical zone for a point source on a surface. This has a radius of many meters due to the longer travel of photons. The intensity of the radioluminescence decreases with an inverse square relationship to the distance from the source (see Figure 3) [5].

**Figure 4:**
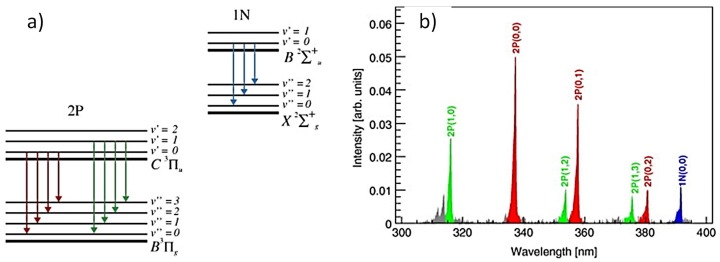
(**a**) Scheme of energy states of the 2P and 1N electronic-vibration band system of $N_{2}$ and $N_{2}^{+}$; (**b**) Nitrogen radioluminescence spectrum between 300 nm and 400 nm in dry air. The same colours are used in (**a**,**b**) for the corresponding spectral bands. Reprinted from [13] with permission from Elsevier.

Although other gasses present in air may also emit UV photons, nitrogen which is the main constituent of air, has been proven to be the main emitter in the 300 to 400 nm wavelength range, in which 95 percent of the radioluminescence intensity occurs [3,13]. Hence, research has been focused on this gas in particular. The radioluminescence has a discrete spectrum as can be seen in Figure 4, which shows the main intensity peaks of nitrogen radioluminescence and their relationship to the 2P and 1N energy states from which they arise. Some gloveboxes may be nitrogen or argon filled as an alternative to air for operational purposes. An increase in the nitrogen concentration has been shown to provide an increase in radioluminescence intensity, likely due to the reduction in oxygen which quenches radioluminescence [14]. Argon may also provide a more intense radioluminescence, though this requires further experimentation and verification.

**Figure 5:**
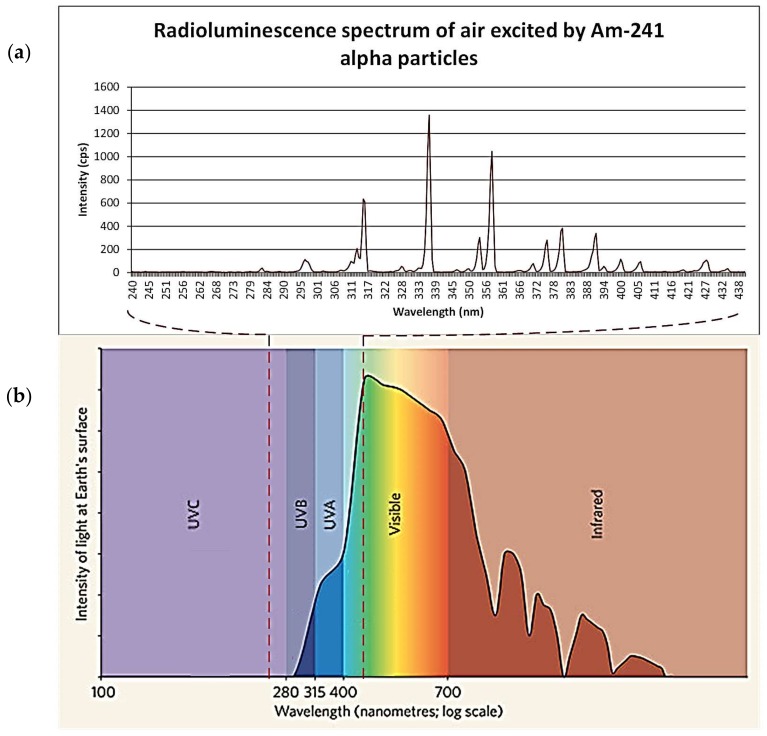
Comparison of the spectrum of alpha-induced photon wavelength in comparison with the spectrum of sunlight at the surface of the earth [15,16]. Image (**a**) produced using data with the permission of the author [15]; Image (**b**) reprinted from [16] by permission from Springer Customer Service Centre GmbH.

Much of the radioluminescence seen in air and nitrogen atmospheres is within the range of solar radiation wavelengths (see Figure 5). The intensity of daylight above approximately 300 nm is far greater than the intensity of radioluminescence due to the presence of an alpha source. Sunlight irradiance in 300 to 400 nm wavelength range reaches (2–8) × 10^−2^ W cm^−2^ nm^−1^, whereas the brightness of the peaks of nitrogen radioluminescence are in the order of 10^−10^ to 10^−7^ W cm^−2^ nm^−1^ for sources within the 3.7 × 10^7^ Bq activity range, and even at night the ambient light will be greater than the radioluminescence signal [3]. This provides a challenge to the detection of alpha-induced radioluminescence where a large background signal is present which must be removed by filtering, working in darkness or avoiding the range of sunlight by working in the UVC wavelength range (180–280 nm).

**Figure 6:**
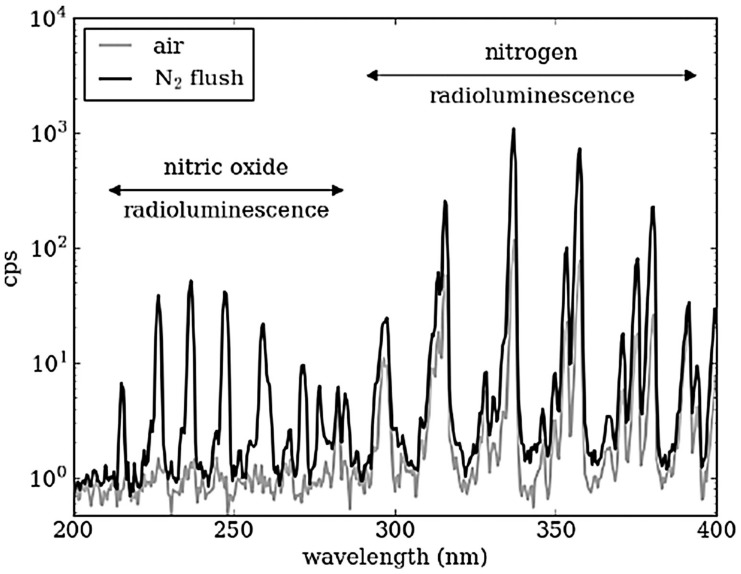
Increase in radioluminescence in the 200–400 nm wavelength range using an N2 purge. Reprinted from [17] with permission from IEEE.

Kerst et al. investigated the effect of nitrogen on radioluminescence in the UVC wavelength range [17]. They note that although molecules in air can potentially emit light of below 300 nm, only N_2_ can produce an amount which is detectable. They therefore tested a ^210^Po source in a N_2_ purged atmosphere and found increased radioluminescence in the sub 300 nm wavelength range due to an increase in NO luminescence, see Figure 6. This increase in radioluminescence in the solar blind region has implications in detection without the interference of background light if replicable in field conditions. However, little effect has been found on the cps recorded by a UVC detector (UVTron, Hamamatsu) when N_2_ was flowed over a ^210^Po source [18]. This may have been due to using a flow rather than a purge.

In calculating the number of UV photons produced in the process of radioluminescence, several results have been put forward, with values ranging from 20 to 400 depending on the number of alpha particles and the energy [3,11,12,19]. Two more recent pieces of work specifically looking at the radioluminescence yield of alpha particles, Sand et al. and Thompson et al. [19,20], were able to correlate their findings with previous cosmic ray analysis of secondary electron radioluminescence, which would seem to verify their results. Sand et al. concluded that there are 19 ± 3 photons per MeV of energy released from the source. This remains linear between 0.3 MeV and 5.1 MeV. Therefore a 5.1 MeV alpha particle would cause the emission of on average 97 photons. From their measurements Sand et al. found the efficiency for conversion to luminescence from kinetic energy was 6.7 × 10^−5^ using 350 nm as a representative wavelength for all photons [19].

Thompson et al. have developed a model which as part of the Geant4 simulation software framework is able to predict the yield of air-radioluminescence photons produced by ionising radiation from alpha and beta radiation sources in the first negative and second positive exited states of N_2_ [20]. Their results are sufficiently close to those found in experimental methods, for example Sand et al. [19], for confidence in the predictive capabilities of the model. Thompson’s model predicted 18.9 ± 2.5 photons per MeV, whereas Sand at al detected 19 ± 3 photons per MeV, showing a strong correlation between results from the simulation and results from observation. Thompson et al. found that a linear correlation existed between alpha energy from sources below 5 MeV and the number of photons produced, also in agreement with existing observations. 

It can therefore be asserted with some confidence that there are approximately 19 photons produced per MeV of alpha energy released from the source.

The energy of the photons produced is linked to their wavelengths which are in turn dependent on the gas in which the ionisation takes place. In nitrogen this is well known and Figure 6 shows the peaks for a nitrogen atmosphere. This is similar to air, where nitrogen is the main component, although oxygen quenches some of the nitrogen radioluminescence, as can be seen by the difference between air and an N_2_ flush.

Other atmospheres have also been tested. For example Grum et al. in research into corona discharge devices identified the emission spectra of corona discharges in nitrogen, helium and air [21]. In a nitrogen atmosphere, they found that in the UVC range it is:

(1) $$h^{\prime }\sum_{u}+n\;\to X,$$

Mechanism that is responsible for the emissions, rather than:

(2) $$C^{3}\pi_{u}\;\to \;B^{3}\pi_{g,}$$

which is the primary mechanism above 300 nm. Below 300 nm they also identified additional lines in the air spectrum that are not in the N_2_ spectrum, possibly from contaminants or CO_2_. In helium the spectrum below 300 nm shows only a weak emission at a wavelength of 249 nm. However, it shows a strong signal at 389 nm, whereas nitrogen shows strong signals at 358 and 337 nm, and medium strong at 316 nm. If a gaseous atmosphere is to be used to enhance the radioluminescence signal, it would therefore appear that N_2_ would be more beneficial than helium.

**Figure 7:**
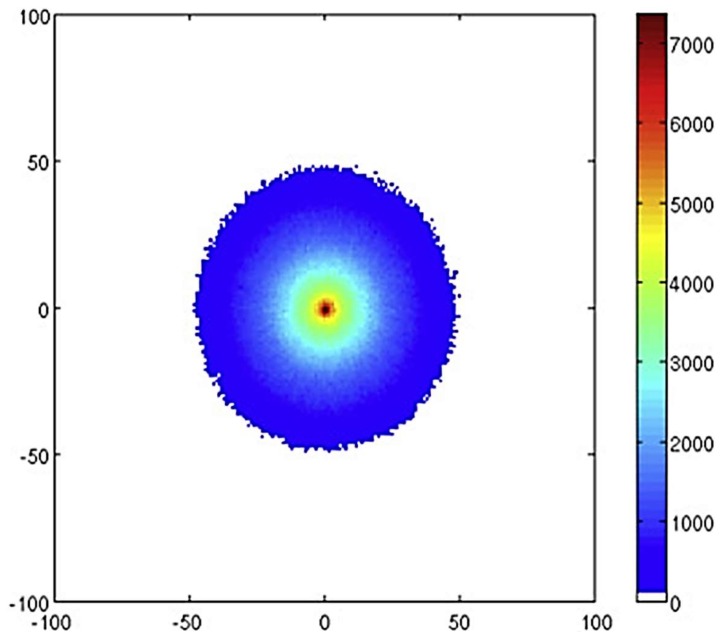
Number of photons emitted per mm^2^ attributed to the 337 nm emission in a 200 × 200 mm area as observed from above a ^241^Am source dispersed over a 2 cm radius circle on an aluminium surface. The ticks give the x and y positions in mm. Reprinted from [20] with permission from Elsevier.

**Figure 8:**
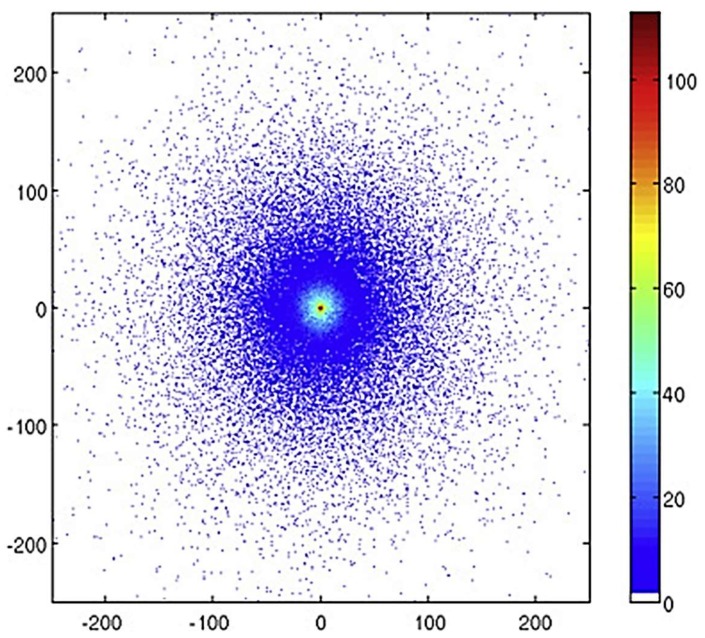
Number of photons emitted per mm^2^ attributed to the 337 nm emission in a 500 × 500 mm area as observed from above a ^60^Co source dispersed over a 2 cm radius circle on an aluminium surface. The ticks give the x and y positions in mm. Reprinted from [20] with permission from Elsevier.

**Figure 9:**
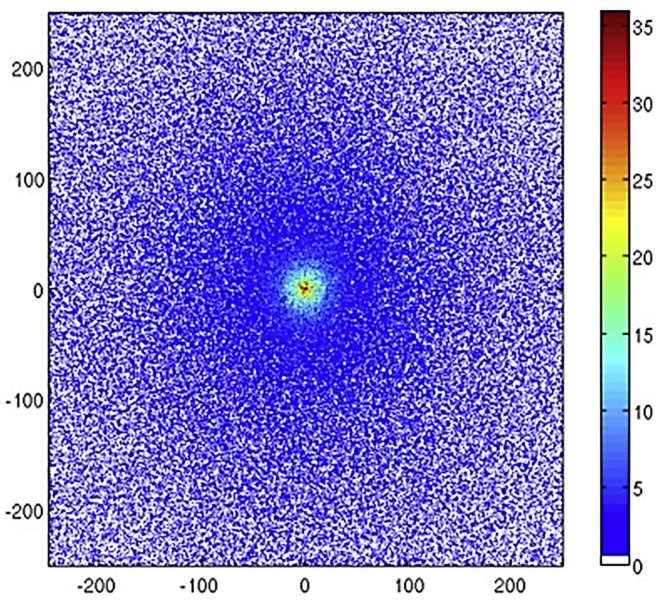
Number of photons emitted per mm^2^ attributed to the 337 nm emission in a 500 × 500 mm area as observed from above a P-32 source dispersed over a 2 cm radius circle on an aluminium surface. The ticks give the x and y positions in mm. Reprinted from [20] with permission from Elsevier.

Thompson et al., alongside developing a model of radioluminescence yield, also investigated the distribution of photons from alpha and beta sources using their simulation [20]. They assert that an alpha source would be easier to locate due to the increased intensity of photons closer to the alpha source. Figure 7, Figure 8 and Figure 9 show how the intensity of photons vary for three different sources, the first (Figure 7) being a 5.48 MeV ^241^Am alpha source of simulated 1 kBq, the other two being primarily beta sources. Although in isolation it would appear that each provide a clear indication of the source location, if considered in a mixed radiation environment where there may be several ionising radiation emitters due to contamination, it becomes clear that the isolation of an individual area of contamination may be more easily accomplished for alpha emitting radioactive sources. Thompson et al. suggest that by measuring the size of the corona it might be possible to estimate the energy of the alpha emission which may provide a means to identify the source material, although the difficulty of isotope identification is discussed later.

It can therefore be seen from the research carried out to date that there are approximately 19 photons produced per alpha emission, 95 percent of which are in the 300–400 nm wavelength range, which is within the solar radiation spectrum at the surface of the earth. The flight of an alpha particle depending on energy is approximately 35–50 mm. Within 10 mm of the source will be the greatest intensity of radioluminescence, with the photons traveling many meters in a spherical or hemispherical pattern, depending on the source geometry.

## 4. Advantages and Drawbacks of Using Radioluminescence

There are several benefits to detecting alpha particle emissions via radioluminescence from ionisation. The main benefit is that detection can be carried out with a greater distance between the source and the detector, reducing detection costs, time and risk to personnel, enabling automated or manual scanning. Photons have a much greater mean free path than alpha particles. In comparison to the 50 mm or so MFP of alpha particles themselves, the induced photons can travel 1 km at 200 nm and 10 km at 280 nm in typical atmospheric conditions [22]. As the photon flux drops off with an inverse square law relationship, the further away from the source that the detector is placed, the more difficult it is to detect the source due to the reduction in signal strength. As UV photons will pass through certain translucent materials detection of alpha contamination can be carried out without breaching containment in instances such as glove boxes or sealed sample bags [4] although modification may be required by the addition of suitable materials.

The radioluminescence phenomenon will always be seen when alpha contamination occurs, and so can be used in all situations. Due to the distribution and reflection of photons, it also does not depend on a line of sight to the alpha source. For example the ‘glow’ may be visible behind an item in a glovebox. This glow can also be imaged and overlaid on a photo of the area in question, which gives a pictorial view of the contamination well suited to analysis by personnel who can then ‘see’ where the contamination is. This image could be analysed for intensity to provide numerical data as well as an image.

Due to the short range of the alpha particles, the photon emissions are relatively local to the source allowing accurate location of the contamination. This also allows differentiation between alpha and other forms of ionising radiation, which occur over a longer range and therefore cause less intense radioluminescence [12]. Researchers found that the ratio of intensities between alpha, beta and gamma induced radioluminescence were 1:10^−8^:10^−10^ respectively, allowing the much greater intensity of alpha radioluminescence to be detected in the presence of other radiation sources [3].

Although theoretically desirable, there are also considerable difficulties with using the radioluminescence approach. The main issue that needs to be overcome is separating the alpha-induced air-radioluminescence from background UV radiation, i.e., sunlight or background lighting. Although the nitrogen radioluminescence has a distinct spectrum, see Figure 4, the main peaks of this spectrum are in the UVA and UVB bands of light (UVA 315–400 nm, UVB 280–315 nm wavelengths) (see Figure 5). Therefore, background light can strongly affect the ability of detectors to identify the relatively weak signal produced by alpha emissions within these wavelength ranges. This inhibits and restricts the use of many of the detectors trialed to date to darkness or carefully controlled lighting conditions, which is unfeasible for most practical decommissioning purposes where a wide range of different environments will be encountered.

UV radiation from the sun in the wavelength range of 200 to 280 nm, known as UVC, is absorbed in the atmosphere by oxygen and ozone [23] therefore there is little background at the earth’s surface in this wavelength range from natural light. Fluorescent lighting also emits very little UV light as this cannot be seen by the human eye and is therefore unwanted. Some fluorescent lamps may emit UVC at 254 nm which is the wavelength at which mercury fluoresces, as this is the mechanism through with fluorescent tubes operate [24]. So there is likely to be some background UV from interior lighting, but little of this will be in the UVC wavelength range for a properly operating lighting system.

Due to the low intensity of the UV radiation from the nitrogen radioluminescence, a high signal-to-noise ratio is required in order to differentiate the signal from any background, and long collection times are needed, in the order of minutes to hours, to reliably detect the signal. Conversion efficiency is the ratio of the energy of the particle transferred to the air during ionisation and the energy converted to radioluminescence. Conversion efficiency figures for the generation of air radioluminescence vary between 1 × 10^−5^ [3] to 6.7 × 10^−5^ [18]; 1.5 × 10^−5^ has been used as a conservative estimate in other work [12].

There are also issues with calculating the exact yield of this radioluminescence. Energy lost to the air has been used to calculate the yield, but due to internal absorption of the source and the complex mechanism of ionisation, it is not always possible to predict yield from a specific isotope via energy lost. As the nature of the radioluminescence does not depend on the isotope emitting the alpha particle, but depends on the energy levels of the gas atmosphere, this makes isotope identification at best complex and at worst impossible using this technique.

In 2013, Roberts [22] looked at the feasibility of using alpha-induced air-radioluminescence for the detection of radiation sources. Through a series of calculations and a number of Geant4 simulations, it asserted that a source with 10^10^ decays per second at a distance of 10 m, would produce a signal of 10 s or 100 s of photons per centimetre square. To verify the presence of the signal suggested by the calculations and simulations, experiments were carried out to detect a polonium-210 source, using a photon counting module and bandpass filter. This verified the emission of photons in the solar blind range by an alpha source, but did not quantify the number of photons in this wavelength range. Although limited in its results in terms of quantifiable experimental data, this work was able to verify the presence of UVC photons and demonstrated an ability to detect these, albeit in dark conditions due to the photomultiplier having some ability to detect photons of above 280 nm. It concludes that it may be feasible to use this method to detect alpha or other ionising radioactive sources, however this would depend on the situation, and that further research would be required, including determining the yield efficiency more accurately for this wavelength range [22].

One other consideration when trying to detect alpha induced radioluminescence photons is the transmittance of UV photons through visible light translucent materials. This is important in both optical elements of any detection system, for example lenses, filters and detector windows, as well as those found in field conditions, for example glove boxes or hot cell windows. The transmittance of a material will depend on the properties of that material and the wavelength of light trying to pass through. All forms of translucent materials have a transmission spectrum which determines how much of each wavelength of light is absorbed or allowed to pass through. This can be tuned by the addition of transition metal, rare-earth ions or nano-crystals to produce band pass filters, which can be useful in blocking out unwanted light. 

Although limited in scope and the number of samples used, Lamadie et al. investigated the transmittance of several materials [5]. They determined that 1 mm thickness of Plexiglas would have a transmission of 91 percent relative to air, 1 mm thickness of polycarbonate would have a 92 percent transmission relative to air and that 1 mm thickness of triplex would have a 91 percent transmittance relative to air. However they do not take into account any specific wavelength differences. The images they present using their detection equipment in the UV range were carried out using a 10 mm thickness of Plexiglas, where they were able to image a source in excess of 1 MBq cm^−2^ at 1 m distance, closer for less active sources. Sand et al. quote attenuation of 80 percent by Plexiglas [10]. However, they do not specify the thickness of the Plexiglas, which they refer to simply as a ‘standard Plexiglas glovebox’. Others have shown various successes at imaging UV photons through Plexiglas, although the images were in the main indistinct [5,25].

In the case of in situ materials, such as glove boxes and detector windows, the attenuation of UV photons can be a significant issue. As part of the research into a stand-off detector several of the researchers have looked into this issue and these results are included in this review.

For full characterisation, not only the presence but also the isotope is required. Although it is theoretically possible for the activity, or at least the emission rate, to be calculated from the intensity of the radioluminescence signal, the wavelength of the optical photons emitted are determined by the gas in which they occur, as opposed to the energy of the alpha particles. As yet, work has not been undertaken on isotope identification, and hence Section 5 looks at alpha particle detectors rather than systems which characterise the isotope.

## 5. Alpha Particle Detectors

This section explores the benefits and drawbacks of traditional detectors which are commercially available, and looks at the prototype and test detectors designed to detect and locate alpha sources through air radioluminescence. Some novel further ideas are also presented. The detectors included are designed to identify the location of an alpha emitter and not to characterise that source, hence carrying out part of the characterisation required for nuclear decommissioning, but not all.

### 5.1. Traditional Detectors

Currently characterisation of sites in regard to alpha contamination is carried out by taking samples which are then analysed in order that the contamination can be identified and characterised. This process takes significant time as samples must be collected and recorded, sent to a suitable laboratory, analysed, and the results returned in a suitable format [9,26]. Therefore it is desirable to have a less time consuming and labour intensive process to locate and identify alpha contamination. The detection of the alpha contamination is traditionally carried out using hand-held alpha radiation detectors.

Although hand-held alpha radiation detectors are readily available, these are in general intended for the immediate detection of alpha radiation for health physics purposes and not characterisation [27]. As these alpha particle detectors, which use a Geiger-Muller tube or more recently a scintillator, work through direct interaction with alpha particles the detector-source distance must be less than that of the range of the alpha particles [3]. This means that the detector must be positioned within a few centimetres of the source in order for alpha radiation to be detected. The benefits of these kinds of detectors are: fast results through the immediate detection of the presence of alpha particles (typically within seconds); good localisation of sources through close proximity requirement; portable; readily available; mature technology.

Although for certain detection purposes this is acceptable, there are drawbacks: proximity to the source provides a hazard for test personnel and requires the use of PPE; detectors may become contaminated if they inadvertently touch the source in hand-held applications; complex plant geometries may make contamination by touch more likely and scanning harder to achieve; time consuming to scan large areas; access issues (limiting penetrations to areas which require characterisation); use in areas of high radioactivity (including safety of personnel, levels of PPE required and contamination of equipment); limited collection of data not suited to isotope identification; no associated automatic mapping of contamination onto an image or map for location purposes.

Hence, it is desirable to find a new method of alpha particle detection which: can be carried out at a distance; is operated remotely; scanning based; completed on site; portable; and possible through clear/translucent barriers (e.g., glove box sides or viewing windows). Therefore, a new way to detect alpha radiation has been sought through secondary effects of alpha particle emissions.

### 5.2. Alpha-Induced Air Radioluminescence Detectors

Alpha-induced air radioluminescence detectors may provide a way forward in overcoming the shortcomings of traditional detectors and there has been significant research in this area in devising a prototype system. Table 1 shows the results of various alpha particle detection research and is included to provide some comparison between the results of different research projects. As can be seen from the table the differences in distances, sources, exposure times, conditions and detector methods makes comparison of the methods and results difficult in determining the most efficient system to date, but some broad conclusions can be made by a comparison in this manner. As of yet, these detectors are designed to locate an alpha source with various success, but identification of the source isotope has not as yet been achieved which would be required for full characterisation.

The remainder of Section 6 looks at this research in more detail, dividing the detectors by technology type.

### 5.3. Solar-Blind Detectors

In order to address the main obstacle to detecting radioluminescence, solar-blind detectors, those sensitive only in the UVC wavelength range, have provided the basis for prototype detector systems shown to be operable in normal indoor lighting conditions.

In 2011 Ivanov et al. used an off-the-shelf, solar-blind, UV camera to locate alpha contamination in daylight conditions through air radioluminescence [30]. They had estimated in 2009 that they would be able to detect alpha radiation of 5 MeV energy with an activity between 40 and 100 Bq cm^−2^ with a corresponding integration time of 600 s to 3600 s from a separation of 3 m between detector and source [29]. The camera they used (DayCor SuperB UV, Ofil Ltd., Lawrenceville, GA, USA) is designed to show the corona and arcing of high voltage equipment for fault diagnosis. It is ‘blind’ to UVA and UVB (400–315 nm and 315–280 nm wavelengths respectively), and only detects UV light of less than 290 nm (UVC). This removes the interference of the stronger background light, allowing detection of the much weaker air radioluminescence in daylight conditions.

They present an image of a 5 × 10^4^ Bq alpha source with an integration time of 10,000 seconds (approx. 3 h). They also present images of background spots generated by noise, as a single frame and a sum of 7500 frames. This shows an apparently random distribution of these background spots over time, which the researchers were able to filter out to some degree for better sensitivity. They also presented a filtered image taken with a 500 s integration time.

Their use of cameras that are available off-the-shelf and are therefore mature technology is beneficial in terms of the reliability. As yet no one has put forward a tested method to quantify the intensity of the light captured by these images, however this could potentially be used to determine the activity levels. This work shows that the approach of using solar-blind detectors in detecting air radioluminescence is viable in addressing the issue of background UV radiation interference, although Ivanov et al. note that there is future work to be carried out to quantify and apply their findings [30].

In 2017 Crompton et al. were able to detect the radioluminescence from a 6.95 MBq Po-210 source from 20 mm distance using a solar-blind UVTron flame sensor (UVTron R9533, Hamamatsu, Hamamatsu City, Shizuoka Pref., 430-8587, Japan) in ordinary laboratory lighting [18]. This sensor is designed to detect the UVC emissions from flames for fire detection purposes and is sensitive in the 180–260 nm wavelength range. The sensor was used with the manufacturer’s off-the-shelf driver board configured to emit a pulse for each UVC photon detected. An average pulse rate of 0.3280 cps was recorded, with a background pulse rate of 2.224 × 10^–3^ ± 0.7034 × 10^–3^ counts per second. A fused silica window was inserted between the sensor and source to prevent alpha particles directly impacting on the sensor. Although the distance between sensor and source is small, they assess that in this configuration the maximum detectable distance could have been 240 mm.

Crompton et al. also tested flowing various noble gases over the source. They found that xenon increased the cps by 52%, P-10 increased the count by 32%, neon by 26%, and krypton 23%. Interestingly they found that nitrogen had little effect on the cps. However, they note that these results require replication for verification, especially in light of the difference between the increase in radioluminescence reported in a nitrogen purge (Hannuksella et al. [14] and Ihantola et al. [4]) with the flow results presented by Crompton et al.

Although the sensor used in Crompton et al.’s research was only shown to work over a short distance in these experiments and its ability for locating the source was not tested, they point out that these initial experiments indicate that this sensor may be viable for stand-off alpha detection if used with other elements in a detector system. This is due to its low background count and insensitivity to indoor lighting conditions. Also, that using a flow of gas which could be achieved through the deployment of a thin flexible pipe, which may be more easily provided in field conditions due to not requiring a gas-tight enclosure and the purging of air, could enhance radioluminescence for detection purposes. This presents a far from developed detector system, but does show a possible sensor which could be used as a foundation for the development of such.

Shaw et al. note the limitations of using PMTs to detect UVC photons, and explore the background and function of new detectors in development, Geiger-mode avalanche photodiode (GM-APD) detectors [23]. This semi-conductor based alternative may make alpha induced air radioluminescence easier to detect than using CCD or PMT. They compared 5 different existing detection technologies, before detailing the GM-APD detector. In their tests this shows a better quantum efficiency at a wavelength of 270 nm (just inside the UVC range). Although their work does not include any testing for alpha detection, this provides an alternative detector technology which may prove useful in the detection of alpha induced radioluminescence. They also explore a number of possible applications of this technology, including the imaging of deep-UV (UVC).

The use of UVC detectors seems to somewhat overcome the issue of background interference from other light sources, however the low signal strength due to the smaller number of photons emitted in this wavelength range is an issue in terms of the distance at which these may work. Others suggest though that solar-blind detectors may not be completely ‘solar-blind’ and hence that the use of external filters to ensure that there is no interference from longer wavelengths may still be required [23] although these would also attenuate the signal.

### 5.4. UVA and UVB Cameras

Other detectors trialed to date specifically focus on the main peaks in the nitrogen radioluminescence spectrum, which occur at wavelengths between 310 and 400 nm, as 95 percent of the intensity falls into this range [3]. Although in this range the number of generated photons is greatest, the intensity of UV radiation from other sources is much higher, i.e., sunlight and traditional artificial light. Therefore, these detectors must be used in complete darkness or with artificial lighting of specific wavelengths, even when filtering or background rejecting methods are used. This limits their practical application.

Work using camera-based systems has mainly focused on locating alpha sources rather than characterising them, with an overlaid image of the radioluminescence over a conventional image being the preferred method of demonstrating the presence of an alpha emitter. This results in images where contaminated surfaces seem to ‘glow’.

Lamadie et al. used a CCD and objective lens to detect alpha sources using radioluminescence [5]. The CCD was cooled with liquid nitrogen and was backlit, which gave it a 60 ± 5 percent quantum efficiency (QE) in the 300 to 400 nm wavelength range. This is in comparison to Sand et al. [10] whose EMCCD achieved a maximum QE of 38 percent in the nitrogen radioluminescence wavelength range.

They noted that the luminescence was visible in what they termed a `bubble’ around the source with an approximate radius of the range of alpha particles emitted from the source, with the intensity reducing relative to the square of the distance from the source. They found these `bubbles’ limited the separation distance between sources at which the two luminescence zones could still be distinguished, which was greater than the resolution of the equipment used, and was between 30 mm and 50 mm depending on the energy of the alpha particles. They were also able to detect bulk contamination, showing that internal absorption that did not fully restrict the emission of alpha particles did not prevent detection.

They developed two equations to calculate the activity of the sample based on the signal intensity and the number of photons per alpha emission, both of which were verified by their experimental results.

The limitation of Lamadie et al.’s work is that it required long integration times of between 1 and 5 h and was carried out in complete darkness. It does however provide advancement in the quantification and characterisation of the radioluminescence phenomenon.

In 2013, Sand et al. tested an EMCCD device to carry out alpha imaging in a glove box with a quartz glass window [32]. They were able to image two mixed fuel pellets (uranium and plutonium), with a 60 s exposure time. The experiment was most likely carried out in darkness as they cite this as being beneficial.

Sand et al. continued with this work in 2015 when they compared the efficacy of two low light cameras; an electron-multiplying CCD (EMCCD) and an intensified CCD (ICCD) [10]. They tested both the differences between the two cameras and also the effect of detecting several sources of different activity at the same time. Their samples were of various alpha emitting materials, and activities ranged from 106 kBq to 4.3 GBq.

Both Sand et al.’s systems are sensitive to natural light (visible and UV) and therefore tests were carried out in near darkness. Testing was carried out in a modified glove box where one of the glove ports had been replaced with a quartz glass window to allow a 90 percent transmittance of photons, as compared to approximately 80 percent attenuation by standard glove box Plexiglas. Their optical results are overlaid on a conventional image.

These images show that although the higher activity sources were detected, those emitting similar radioluminescence intensities to the low background light were undetectable to both systems. They were able to achieve a resolution of better than 1 cm between sources. They also found that high intensity sources could mask lower intensity ones and suggested re-imaging after the removal of high intensity sources to check for sources of lower intensity, using longer exposure times or reduced background lighting. Sand et al. conclude that the ICCD gave marginally better results in the field than the EMCCD, partially due to its greater field of view.

Pineau et al. (patent registered) put forward a proposed stand-off alpha detection system which is broad ranging in its description, and as such all avenues of operation it describes may not necessarily have been shown to work [35]. Their main assertion is to fill the environment containing the source with a scintillating gas, which may contain nitrogen. As nitrogen has been shown to be the main radioluminescence emitter in the UV range, this is consistent with other findings. This could be in an enclosure which is placed over the area to be investigated, which will retain the scintillation gas and has a window transparent to UV photons. However, the flow of gas used in other work [18] could be easier to apply in the field than the need for a gas-tight enclosure to be deployed in potentially difficult to access or contaminated areas.

Pineau et al.’s detector is described as being a CCD type detector, connected to a ST 138 type controller. Due to the small number of photons produced, the system will integrate a number of images, therefore increasing the detection time. They suggest using a wavelength range of 200–400 nm. The device may also have a camera able to take a visual image over which to overlay the image of the alpha induced photons. Due to the possible interference of light in the visual spectrum, they suggest using the system in darkness or using filters to attenuate light outside of the UV spectrum. No results are presented in the effectiveness of this system, however, for a patent to be applied for it may be assumed that they were confident that this system would work and therefore that tests had been successfully carried out.

Haslip et al. use a comparison of the alpha induced nitrogen radioluminescence signals of four wavelengths; two wavelengths where nitrogen radioluminescence peaks, and two where it does not which present the background signal [36]. A telescope is used to collect the signal, which is amplified by mirrors and focused on six UV-sensitive cameras. This is achieved through the use of beam splitters and wavelength selective filtering. Images from these 6 cameras are collated by a microprocessor proving an aggregated image to the operator which is in almost real time. Although this system is not able to reject daylight, it can be used at night where these is still a significant amount of background UV radiation, or under street lighting.

In 2008 Giakos proposed a stand-off alpha detector architecture using a spectrometer and ICCD camera, with a focusing assembly of lenses and reflectors [28]. Their calculations indicate that two 3.7 × 10^7^ Bq ^239^Pu sources could be detected at 25 m, even in the presence of an 18.5 × 10^7^ Bq ^60^Co gamma source. They also suggest that the use of an active system using a Raman lidar system along with the passive radio-luminescence detector, would not only be able to determine the presence of a radiative source, but also indicate it’s biological hazard by determining the energy loss associated with the detected light though the specific spectrum. The calculations are presented in the research paper to show how the architecture was devised, but there is no evidence that this system was tested and therefore if it was successful or not, or any limiting factors found during any experimental trials.

### 5.5. UVA and UVB PMT Based Detector

Due to the ability to more easily quantify the signal intensity, other prototypes utilise a PMT to detect the radioluminescence. In 2010 Leybourne et al. reported their prototype detector was capable of detecting a Po-210 source (37 MBq) at 150 m distance from the detector, outdoors [31]. Using optical filtering, telescope optics for collection, and a PMT (photo-multiplier tube), they were able to detect the presence of an alpha emitting source on the surface of any one of three, 55-gallon drums spaced 10 m apart at approximately 150 m distance. This was achieved in less than 1 min of data acquisition time for each source. Although not specifically stated it can be inferred from the text that these experiments were carried out at night as there is reference to ‘heavy traffic’ and ‘other surrounding outside illumination’ causing interference. However, even at night there is significant UV radiation outdoors.

Leybourne et al.’s filtering was able to attenuate background UV radiation and provide a sufficiently high signal-to-noise ratio to differentiate the relatively weak UV radioluminescence. They also noted an inverse squared relationship between the intensity of the UV photon signal and distance, as would be anticipated in a spherical (or hemispherical) isotropic photon emission zone around a point source.

The result of Leybourne et al.’s work is very positive in terms of indicating that it is possible to detect alpha emissions through air radioluminescence in the presence of significant UV background. However there are several drawbacks and limitations to the work. A relatively crude approach was taken for identifying the alpha source, in terms of a resolution of 10 m between sources (i.e., the distance between the drums) and the variability of the counts which show little more than the presence of a single or double source rather than anything about the nature of the source. It is possible that the experiments were carried out at night, to reduce the background UV that the device was required to reject. There is little information on the equipment specification or models used to carry out the experiment, meaning that it could not be replicated to check the accuracy of the work. This includes the bandpass of the filtering system. However, whilst limited this work does show that there are approaches to this method of alpha particle detection which may prove viable in the field.

Baschenko used a monochromator and PMT in photon counting mode to determine the spectrum, and low light sensitive film to image the source [3]. They found that the ratio of intensities between alpha, beta and gamma induced radioluminescence were 1:10^−8^:10^−10^ respectively, allowing the much greater intensity of alpha radioluminescence to be detected in the presence of other radiation sources. This has two implications. The first being that this technique can be used to combat exposing personnel to beta and gamma radiation, which may also be present within the range of traditional alpha particle detectors. The second is that the different types of radiation do not interfere with the alpha detection, making it suitable for mixed radiation environments normally seen within the nuclear industry.

Whilst characterising the alpha induced radioluminescence, Baschenko found that 95 percent of this was in the 310 nm to 400 nm wavelength range and was due to the 2^+^ nitrogen transition system. They calculated that there were approximately 30 UV photons emitted per alpha event, with 2.5 × 10^−5^ of alpha particle energy being transformed to photon energy. They also assert that alpha particles may be emitted in a cone shape with an angular distribution which is proportional to $\;\cos{\;}^{8}\left(\theta \right)$, where *θ* is the angle between normal to the surface and the flight of the alpha particle. Although this conclusion is not supported by other literature which finds the emission of photons is isotropic [19] and therefore is likely to be a misinterpretation or anomaly in the results.

Baschenko used these results to calculate a possible detector set up. From calculations of the effectiveness of this system, they were able to determine that this would not be suitable for use out of doors as background UV would always exceed the required level, even at night.

Other work of Sand et al. focuses on two potential methods of detecting radioluminescence; spectral and coincidence filtering. In 2010 Sand et al. and Hannuksela et al. tested both these methods [11,14]. They compared background lighting to the radioluminescence signal using a beam splitter and interference filters in a device they named Handheld Alpha UV Application (HAUVA).

Noting that cameras require relatively long integration times, Sand et al. and Hannuksela et al.’s spectral filtering detection system uses two PMTs, which allows detection using an integration time of approximately 1 s for a 100 kBq source at 400 mm distance from the detector. This was achieved under artificial background lighting conditions which did not produce UV. Using a 40 nm bandpass filter, the signal was first filtered into the peak air radioluminescence wavelength range, 300–340 nm (where 337 nm is the most intense peak of the spectrum). The signal was then split, with the background portion being passed through a further 15 nm bandpass filter giving a 299 to 303 nm wavelength range.

Using two PMTs and a time correlated single photon counting unit Sand et al. and Hannuksela at al. verified that all photons from a single alpha decay were emitted in one 5 ns time window, as found in earlier work. This time period was sufficiently short to make a background count event at the same time as an alpha induced photon improbable. Using coincidence filtering, they were able to detect radioluminescence against background light which was 500 times more intense than the radioluminescence. At this stage in their work, they quote a value of 400 photons per 5 MeV alpha emission. However this is reduced in later work to 20 photons per MeV of alpha energy, more in line with others’ findings.

Sand et al. and Hannuksela et al.’s optimised optics, designed with a large collection angle to collect the greatest number of emitted photons, have a collection efficiency of 0.12 percent at 400 mm, and they noted how this dropped off rapidly from 300 mm onwards, showing the importance of distance to source. They also found a rapid drop in signal intensity when the source was moved 20 mm to the side, giving a positive indication for source location possibilities.

By using a nitrogen-only atmosphere and a 10 kBq ^241^Am source, Sand et al. and Hannuksela et al. found that the detector counts per second increased to 650 cps, from 150 cps in normal atmosphere. They attributed this increase to the removal of the quenching effect of oxygen.

Building on their earlier work, in 2016 Sand et al. published the results of alpha induced radioluminescence detection experiments carried out in bright lighting conditions [16]. Using the same set up with two different equipment options, they were able to distinguish a 4 kBq source at 1 m in 10 s under UV free lighting, and 800 kBq under bright fluorescent lighting.

The general set up for Sand et al.’s experiments comprised of a telescope, utilising two lenses to focus photons onto the eyepiece. This light passes through a filter stack before being focused onto the window of a PMT. The PMT is used in photon counting mode to determine the intensity of this signal. Two different filter stacks and PMTs are used. The first is a PMT with an ultra-bialkali photocathode which is sensitive in the near UV range. The associated filter stack is sensitive at a central wavelength of 335 nm. This was tested under yellow lighting conditions. The other set up utilises a solar blind PMT which has a caesium-telluride photocathode, with a filter stack centred at 260 nm, which was tested under fluorescent lighting conditions.

Sand et al. recognize the limitations of their systems, in that they are not suited to imaging due to utilising telescope optics, and that scans are time consuming due to the narrow field of view. Due to the differing field environments, each site would have to be surveyed in advance to determine if these detector systems were suitable for that particular site. They also note that solar blind camera detection methods can only be used in open spaces, however, the reasoning behind this statement is not qualified.

Kume et al. build on the work of Lamadie et al. [5] and Chichester and Watson [12], whom they consider to have both developed ‘convenient’ systems for stand-off alpha detection, by addressing the issue of noise generated by a high gamma radiation background which create a low signal to noise ratio [34]. They note that Ihantola et al. have gone some distance in noise rejection by using time-coincidence, but that this has not completely removed the background noise generated by gamma-rays [4]. Their solution is an ‘alpha camera’ which utilises a lens and mirror to focus the UV photons onto the UV detector, a PMT with a response in the range of 300–650 nm, peaking at 350 nm (35 percent QE). Lead shielding around the PMT and mirror reduces the influence of gamma-rays on the system. A CCD camera, also within the confines of the lead shield, provides a visual image over which the results of the PMT can be overlaid to provide a visualisation of the alpha contamination’s location.

One limitation of Kume et al.’s work is that this detector currently works exclusively in dark conditions. Their proposed resolution to this issue for field operations is to use a coating on the lens of their system to filter visual light. There is no discussion on the difficulties that this may present due to the attenuation of the UV light that is likely to occur, or to the wavelength range of the light attenuated by the filter, or what the nature of this coating will be. In practice this may be a more significant issue than they suggest.

Inrig et al. used a position sensitive PMT with UV filters and a series of 6 lenses to detect a 1 µCi (37 kBq) source from 1.5 m distance with a 10 s integration time [8]. This was accomplished in a windowless room with dim lighting by using an algorithm and the known frequency of oscillation of the electricity supply to the lighting in their experimental environment to reject any unwanted light. They were able to image the alpha sources, although the resolution of the images was poor. This method may be suited to internal environments without windows where the frequency of electronic supply oscillation is known. However, it is possibly not well suited for general field operations.

In 2012 Ihantola et al. used coincident spectrometry of gamma radiation and alpha-induced radioluminescence to enhance alpha detection in areas of high activity [33]. Radioluminescence photons from an alpha emission trigger the operation of a gamma detector. Hence only gamma photons which occur in the presence of alpha induced photons are detected. This ensures that the detector is focused on the alpha emitter and not other gamma emitting sources which may produce photons of a higher energy than the alpha emitter and so mask the alpha source. This was undertaken not only to locate the source, but also to characterise the source and determine the isotope, which cannot as yet be achieved with alpha radioluminescence alone. The alpha detector, comprised of a collection lens and PMT, was able to identity a 4.2 kBq source from 157 mm away in both a nitrogen or air atmosphere. In nitrogen the intensity of the signal was 150 times the background, in air 30 times. The 50 mm field of view provided by their test equipment means that a very localised analysis can take place of the alpha emitter. It is also possible to detect sources in sealed containers if the material of these is transparent to UV radiation, where UV photons will escape, but alpha particles will be stopped by the container.

Their experiments were carried out in the dark, and Ihantola et al. suggest the use of filters for daylight working. Although the system worked, they conclude that the UV system was better for locating the source and the gamma detector for identifying the isotope, and suggest the two could be separated for better efficiency. In 2013 this work was continued using dim red LED lighting which allowed a level of illumination sufficient for working and for imaging of the set up [4]. They note during this work that the coincidence filtering method works better with a high gamma background and integration times of minutes or hours. This method allows an avenue for the identification of the isotope as the gamma emissions are more suited to this type of analysis than alpha induced radioluminescence.

There are limitations to this work of Ihantola et al. Detection of the alpha-induced radioluminescence photon suffers from the same issues as with other detectors, primarily the interference of environmental light sources. However, Ihantola et al. found that this coincidence spectrometry technique is ten times faster than a conventional gamma spectrometer, and from this it seems that their assertion that it is a step forward is not unsubstantiated.

All of the above research results confirm that it is possible to detect alpha induced radioluminescence in a number of ways and situations, but as these require a background of no, low or special light they are unsuitable to be used in the field at present due to the difficulties in controlling the lighting conditions.

### 5.6. Other Detector Types

Although in the main recent detectors have focused on the detection of nitrogen radioluminescence photons, this is not the only possible secondary effect of alpha particle emissions which could prove suitable for the detection of alpha contamination.

Sprangle et al. put forward an alternative method of stand-off radiation detection through the use of an ionising laser and a probe beam [37]. Although their work is specifically for gamma detection, they plan to test their concept using an alpha source to reduce the safety issues. Hence this method may be suitable for alpha detection. Ionising radiation produces free electrons in air. These attach themselves to oxygen molecules and form O_2_ ions in greater concentration to free electrons. A high powered laser, focused close to the radiation source is used to photo-detach the negative ions, which initiates an avalanche ionisation process. A probe beam can then be used to detect the changes in electron density caused by the avalanche ionisation, and the presence of radioactivity determined using measurement of the frequency modulation. The main advantage of this system is that it would be able to detect ionising gamma radiation from distances greater than 100 m.

Sprangle et al.’s paper highlights a potential design concept for a gamma detector, which has two drawbacks when applied to alpha detection. This is a design concept which has not as yet been proven for gamma detection for which it was designed. In addition, an examination of the possible feasibility of using this design for alpha detection is not presented in this paper. For example the much shorter mean free path in air of alpha radiation in comparison to gamma radiation is likely to produce a smaller ionisation ‘bubble’ which may present challenges in focusing the laser sufficiently close to the alpha source without prior knowledge of its whereabouts. It may also find the materials used for shielded windows challenging, for example in glove boxes or hot cells. However, this does present a possible alternative method of alpha detection possibly at further distances, which may merit further consideration.

In order to address the propagation loss at a distance from the source to the detector, Yao et al. used a collimated beam emission from a nitrogen laser at a wavelength of 337 nm to further excite alpha ionised air molecules from the $B^{3}\pi$ to $C^{3}\pi$ state [38]. The absorption of the energy required was detected and from this the presence of alpha radiation was identified. This detector was successful in detecting a 1.48 GBq source at a maximum standoff distance of 10 m. They found that the detection signal was not sensitive to the distance between the detector and source, as it is with the photon detector methods. In their tests they were able to determine the relative intensities between two sources of different activities. They also note that due to the longer carrier lifetime in the $B^{3}\pi$ band compared to the $C^{3}\pi$ band, the population of carriers in the $B^{3}\pi$ may be an indication of the intensity of the radiation causing the excitation.

**Figure 10:**
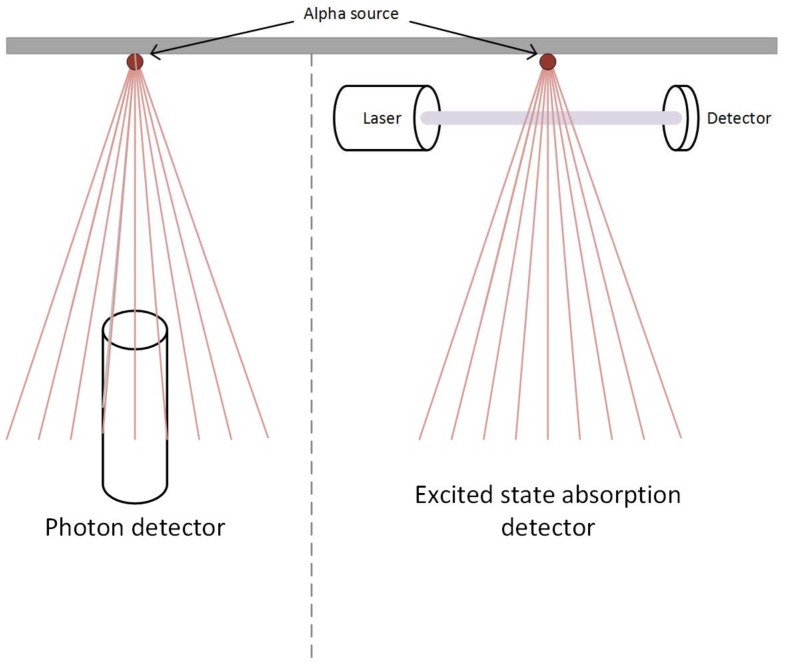
Deployment of photon v. excited state absorption detector systems.

Although the work of Yao et al. was successful in identifying the presence of an alpha source its main drawback is the required detector configuration. It requires an emitter and detector diametrically opposite each other in line with the alpha source. This means that both sides of the alpha source need to be accessible, which may not be possible for surface contamination, or in other hard to access areas. It would make scanning difficult to conduct, as the detector alignment would need to be parallel to any source, rather than perpendicular (see Figure 10). As the distance from the source to the laser or detector has no effect on the signal it would not be possible to determine the position of the source between the two, and direction to the source would be difficult to determine. Hence, it would be difficult and time consuming to find the source of the alpha emissions.

Baschenko suggests a similar alternative method, using a laser of specific wavelength which would affect air molecules already excited to a certain energy state due to alpha ionisation [3]. This is the same as they approach of Yao et al. [38], but Baschenko aims to detect the change in the number of photons that are emitted due to the increase in energy created by the addition of the laser energy to the already excited nitrogen molecules, rather than changes to the laser probe signal. Baschenko has not tested his approach and merely mentions that this may be theoretically possible, whilst noting that there would be significant technical difficulties in using this approach.

Allander et al. developed a system for detecting the ion pairs produced by alpha particle ionisation of the surrounding air, which they call the LRAD system (Long-Range Alpha Detector) [39]. It utilises an air current or an electric field to transport the ion pairs to a collection grid where they are detected as an electric current, the current being proportional to the activity and therefore allowing a measurement of this. However, these require either that the potentially contaminated object is placed inside a chamber where filtered air can be flowed over it to carry the ion pairs to the grid, or for the detector system to be introduced into an existing pipe where an air flow can be used to measure any contamination inside the pipe. Both of these have implications for the ease of use in the field, and the initial setting up of the system, including moving and cutting into potentially contaminated materials. A third method allows for the detector to be placed over a potentially contaminated surface (for example soil or a concrete floor) and an electronic field be used to detect the ion pairs. The main drawback of this system is that the detector could come into contact with contamination, thus becoming contaminated itself, and still requires the operator to be in close proximity to the contamination to set up the device. However, in processing samples, especially in large quantities, and for internal pipe examination, these methods could prove superior to traditional techniques. Certainly radioluminescence would be harder to detect within a pipe without special deployment equipment.

## 6. Future Prospects for Alpha Induced Radioluminescence Detection

Initial work in the detection of alpha contamination through nitrogen radioluminescence has concentrated on the main peaks of the radioluminescence spectrum, which occur in the 300 to 400 nm range. This leads to background UV radiation from the sun or artificial lighting interfering with the detection of the alpha induced radioluminescence by masking its much weaker signal. Filtering of the wavelength of photons detected allowed for the imaging of alpha sources in dark or special background lighting conditions, but not as yet in daylight. By moving away from the UVA and UVB range into the UVC range a possible route to overcoming this limitation becomes apparent. Although the peaks of intensity in this band appear to be lower, there is not the competition from sunlight and artificial light, improving the signal to noise ratio. This would potentially make detection possible on site in nuclear installations to provide characterisation for decommissioning and other purposes.

A detailed analysis of the spectrum of UVC is required, including identification of any significant peaks which may provide the best chance of detection. Other gasses may provide a better scintillation atmosphere, including in the UVC wavelength range and should be investigated. Tests carried out in the 1960s provide some information regarding the effect on wavelength of emitted light in various gas environments, for example, see Morse et al. [40]. However, these require further investigation to apply them to enhancing the scintillation for specific required wavelengths.

Other beneficial future work would include the further testing of UVC/solar blind detectors to determine their efficacy in detecting alpha induced radioluminescence. A review and testing of currently available UVC detection technology would allow an assessment of it this could be utilsed to develop a new UVC detector specifically for nuclear decontamination purposes.

Putting together a number of effective techniques to provide a multi-stage detector may be the route forward. These other possible techniques include but are not limited to: data processing algorithms, collection optics, superposition and amplification, and the use of light reactive materials. A multi stage detector may provide a more efficient and robust detector for use in the field. Coincident and background attenuation techniques are the subject of continuing experimentation and could be expanded, as could active detectors of the kind as put forward by Baschenko and Yao [3,38].

Transmission through translucent materials for different wavelengths requires more investigation for a completely suitable field detector to be produced. The limited research carried out to date does not contain sufficient detail or analysis of the phenomenon to determine how much of an issue this will be for detection in the field, and how this can be addressed. Tests to show both the internal and external transmission would be useful, for conditions where the surface reflection of the glass may or may not be relevant. In lenses and filters the internal transmission is more relevant as an anti-reflective coating can be used. This may not be possible for gloveboxes and hot cell windows, hence the external transmission may be more suitable. It may also be beneficial to test transmission of existing materials in the field where the age of the materials may also prove influential as some of the nuclear sites for decommissioning are of a substantial age. An understanding of the transmission of these materials may also be beneficial in determining if contamination is on the interior of the translucent material or at a distance which has not as yet been addressed, most likely due to researchers already knowing the location of the contamination in test situations.

Although there is a great deal of existing research and information, the differences in distances to source, detectors, sources and other conditions makes an assessment of progress difficult. A systematic testing regime with single variable differences between tests would provide a more easily accessible and comparable set of results, in terms of effect of yield on different conditions (gasses, translucent materials, reflection etc.) and the efficacy of different detector types.

Work to date has provided a sound basis for continuation, with a clear route along the UVC wavelength path, possible benefits from the identification of an alternative radioluminescence gas, and routes using optics and other methods to optimise the collection, processing and detection of alpha-induced air-radioluminescence photons. This work will lead to the development of an alpha detection system that can be used on site for nuclear decommissioning purposes.

## Figures and Tables

**Table 1:** Summary of alpha particle detection research to date.

Authors Year Ref.	Distance Time Activity (Where Known)	Source	Equipment	Conditions
Baschenko 2004 [3]	30 m, 3.7 × 10^7^ Bq	^239^Pu	Monochromator & PMT for spectrum Reflector & film for image	Darkness Presence of strong gamma source
Lamadie et al. 2005 [5]	1 m, 600 s, <1 Mbq. cm^−2^	^244^Cu	CCD, Fused silica objective lens	Through 10 mm Plexiglas, Darkness, Field Test
	10 cm, 3600 s, 30 kBq	^241^Am	“	
	20 cm, 5 h, 3.88 kBq	^238^Pu	“	
Giakos 2008 [28]	25 m, two 3.7 × 10^7^ Bq sources	^239^Pu	Spectrometer, ICCD camera, lenses, reflectors	Theoretical. Daylight. In presence of 18.5 × 10^7^ Bq ^60^Co gamma source.
Ivanov 2009 [29]	3 m, 600 s, 10^5^ Bq	5 MeV alpha emitting, point source	“	Estimate of performance in daylight
Ivanov 2011 [30]	10000 s, 5 × 10^4^ Bq		DayCor UV camera	Daylight
Leybourne et al. 2010 [31]	150 m, <1 min, 5 mCi (185 MBq)	^210^Po	PMT, Filters	Field experiment
	150 m 1 mCi (37 MBq)	“	“	Theoretical
Sand et al. 2010 [11]	0.4 m, 1.2 kBq, 620 cps	^239^Pu, ^241^Am, ^244^Cm	HAUVA (own design)	Spectral filtering
	0.4 m, 1 s, 100 kBq		“	Yellow radioluminescence or white LED light
	0.4 m, 1 s, 1 kBq	^241^Am	„	‘Selected room lighting’
	0.2 m, 13 kBq	„	„	Coincidence filtering
Sand et al. 2013 [32]	>1 s	uranium, plutonium	EMCCD	Darkness
Sand et al. 2015 [10]	0.5 s, 0.52 GBq	Pu nitrate	„	Darkness, glovebox, quartz window
	30 s, 4.0 GBq	Mox pellet	„	„
	100 s, 0.52 GBq	Pu nitrate	„	„
	100 s, 52.79 MBq	^239^Pu plancette	„	„
Sand et al. 2016 [16]	1 m, 10 s, 4 kBq		PMT, optics, filter stack	UV-free lighting
	1 m, 10 s, 800 kBq		„	Bright fluorescent lighting
Inrig et al. 2011 [8]	1.5 m, 10 s, <37 MBq	^241^Am	PMT, filter, optics	Artificial light (60 Hz)
Ihantola et al. 2012 [33]	0.157 m, 4200 Bq	^241^Am	PMT, filter, optics	Light tight box, nitrogen atmosphere
Ihantola et al. 2013 [4]	0.1 m, 50 Bq	^241^Am	„	Red LED lighting
Kume et al. 2013 [34]	1 m, 30 s, 1.5 kBq	^241^Am	PMT, lens, mirror	Darkness
	1 m, 20 s, 9 kBq		„	LED light–centre wavelength 635 nm
	1 m, 30 s, 1.5 kBq			Theoretical
Crompton et al. 2017 [18]	20 mm, 6.95 MBq	^210^Po	Solar-blind UVTron flame sensor (Hamamatsu UVTron R9533)	Ordinary laboratory lighting.
